# Biphasic Effects of Naloxone in the Rats Receiving Morphine Overdose A Place Preference Study

**Published:** 2011

**Authors:** Sara Karimi, Manizheh Karami, Homeira Zardooz, Seyed Hassan Salimi, Hedayat Sahraei

**Affiliations:** a *Department of Biology, Faculty of Sciences, Shahed University, Tehran, Iran.*; b *Department of Physiology, School of Medicine, Shahid Beheshti University of Medical Sciences, Tehran, Iran.*; c *Department of Psychology, School of Medicine, Baqiyatallah (A.S.) University of Medical Sciences, Tehran, Iran.*; d *Neuroscience Research Center, Baqiyatallah University of Medical Sciences, Tehran, Iran.*

**Keywords:** Morphine, Naloxone, Sniffing, Rearing, Locomotion, Conditioned, Place preference, Rat

## Abstract

Downward phase of dose-response morphine converted U shape curve was chosen as a base for investigating the effects of different doses of naloxone (0.05-0.4 mg/Kg) on morphine reward/aversion properties using place preference method.

First, male Wistar rats (200-220 g) were received morphine (1-7.5 mg/Kg) for place conditioning and marginal dose of morphine (5 mg/Kg) calculated by GraphPad software. In the next part, the animals received different naloxone challenge doses (0.05-0.4 mg/Kg; IP) on the test day. Animals’ behavior was monitored using a video camera during the test session. Time spent in each compartment was calculated as the main sign of drug seeking behavior. In addition, numbers of rearing and sniffing as well as locomotion activity for each animal were counted as important dopamine-dependent behavioral signs. More over, the total compartment crossing by each animal as the sign of decision making was also counted.

Our results indicated that naloxone showed biphasic effects on the appearance of morphine-induced place preference. The antagonist potentiates the expression of morphine-induced place preference on the dose of 0.05 and 0.4 mg/Kg while inhibits the morphine effect on the dose of 0.1 mg/Kg. On the other hand, the total animal sniffing, rearing, locomotion, and compartment entering were not significantly changed among the groups.

It could be concluded that the inhibition of opioid receptors may enhance or inhibit the expression of morphine reward according to the naloxone dose, which in turn indicate the influence of several opioid receptor in this regard. In addition, opioid receptor blocking did not enhance the signs of drug seeking behavior linked to the activity of mesolimbic dopamine system.

## Introduction

It is now clear that mesolimbic dopamine system which originates from ventral tegmental area (VTA) of the midbrain and projects mainly to the nucleus accumbens (NAcc), is the major target for morphine reward (1, 2). Studies by Johnson and North (3) have indicated that the inhibitory influence of gamma-aminobutyric acid-ergic (GABAergic) interneurons on VTA dopaminergic neurons is removed by mu-opioid receptors activation (by morphine-for example) which actually leads to an increase in extra-cellular dopamine concentrations in the targets of these neurons in nucleus accumbens (3, 4, 5). 

One reliable and relatively simple method for studying opioid reward is the conditioned place preference method (6). Previous studies have indicated that opioids can induce place preference in different animal models (6). Studies indicated that in this method, opioid dose-response curve has an inverted U shape as the dose of the drug increased gradually (For rev see: 7). Activation of different opioid receptors in the VTA is considered as the main reason for such inverted U shape dose-response curve (8). Opioid place preference consists of two distinct components, namely acquisition and expression (6, 9). Understandings of the mechanisms involved in each component were the aim of intense investigations during past years. For example, it is now clear that opioid place conditioning is based on dopamine receptor activation in nucleus accumbens (10, 11, 12). In addition, several neurotransmitters in different parts of central nervous system have also been shown to be involved both in the acquisition and expression of opioid place preference (See 6 for Rev). Interestingly, it is not clear if opioid receptors are also involved in the expression of opioid place conditioning or not. In order to answer this question, the present study was designed. For this reason, we first determined morphine (as typical opioid) dose-response curve, then calculated the downward point of the curve and finally the effect of naloxone (as opioid receptor antagonist) on this point was studied. Naloxone which was chosen as the antagonist can inhibit different opioid receptors in different doses i.e., in lower doses it inhibits mu-opioid receptors and in higher doses it inhibits delta-opioid and kappa-opioid receptors (13). If the antagonist could change the downward point, one can conclude that opioid receptors are also involved in the expression of morphine and other opioid place preference. 

## Experimental

The male Wistar rats (250 ± 20 g, Pasteur Institute, Tehran, IRAN) were housed in groups of 4 rats per cage in a 12/12 h light cycle (lights on, at 07.00 a.m.), with *ad libitum *food and water available. All experiments were conducted in accordance with standard ethical guidelines and approved by the local ethical committee (The Baqiyatallah (A.S.) University of Medical Committee on the Use and Care of Animals, 81/021, July 10, 2002). 

*Drugs*

Morphine sulfate (TEMAD Co., Tehran, IRAN) and Naloxone hydrochloride (Sigma, USA) were dissolved in sterile saline and were injected subcutaneously in a volume of 1 mL/Kg. For evaluating the effects of morphine on place preference behavior, different doses of morphine (1, 2.5, 5, and 7.5 mg/Kg) were injected into the animals.

*Apparatus*

A two compartment conditioned place preference (CPP) apparatus (30 × 60 × 30 cm) was used. The apparatus was made of wood. Both compartments were identical in size (the apparatus was divided into two equal-sized compartments by means of a white removable wall) and shading (both were white), but distinguishable by texture and olfactory cue. To provide the tactile difference between the compartments, one of them was chosen with a smooth floor, while the other one had a white nylon mesh floor. A drop of menthol was placed at the corner of the compartment with a textured (nylon mesh) floor to provide the olfactory difference between the compartments. Their walls were differently black-striped (one compartment horizontal and the other one vertical) on their sides. In this apparatus, rats showed no consistent preference for either compartment (14). A video camera located 120 cm above the apparatus, recorded all animals’ behavior during the experiments. The video types then were observed by an observer which was unfamiliar to the experiments. 

*Experimental procedure*

Each conditioning session consists of 5 days. On the first day of experiments, each rat was placed separately into the apparatus for 10 min, with free access to all compartments and the time spent by rats in each compartment was measured. In the second phase which consisted of a 3-day schedule, animals received three trials in which they experienced the effects of the morphine while confined in one compartment for 45 min and then three trials in which they experienced the effects of saline while confined in the other compartment for 45 min. Access to the other compartments was blocked on these days. On the 5^th^ day (the preference test day) the partition was removed, and the rats could access the entire apparatus. The mean time for each rat spent in either compartment during a 10 min period was determined as the preference criterion. Naloxone was injected 30 min before the tests in the preference test day.

*Statistical analysis*

Data were given as the mean ± SEM for 6-8 animals. One-way analysis of variance (ANOVA) followed by Tukey’s test were performed to assess specific group comparisons. Differences with p < 0.05 were considered statistically significant.

## Results and Discussion

*Morphine place conditioning behavior in the rats*

**Figure 1:**
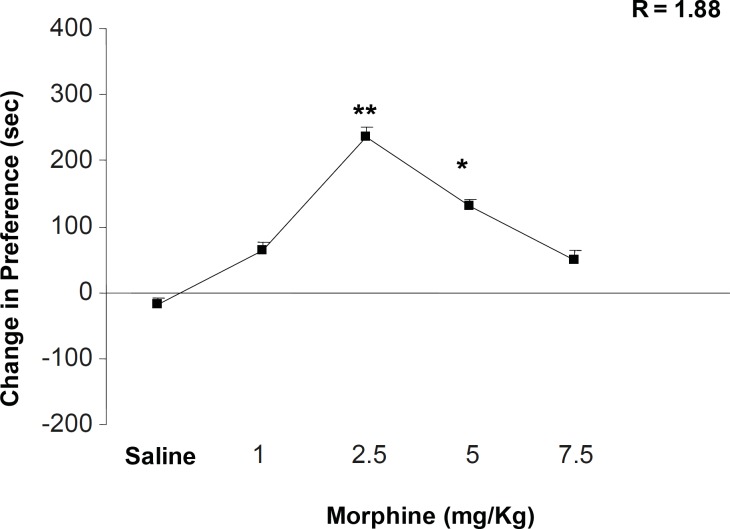
The effects of different doses of morphine on the place preference paradigm behavior. Animals received morphine (1, 2.5, 5 and 7.5 mg/Kg; SC) during conditioning session and were examined in the test day in drug free state. Non-linear regression was also applied for the calculation of ED50 and the opposite point was calculated as downward point. Data are shown as mean ± SEM for 6-8 rats, *p < 0.05, **p < 0.01 different from the saline control group

Figure 1 shows the effects of different doses of morphine on place conditioning behavior. Morphine administration can dependently induce a clear place conditioning dose with maximum effect on 2.5 mg/Kg of the drug [F (5, 38) = 4.352, p < 0.001]. In addition, non-linear regression calculation indicated that half-maximal effective dose (ED50) of morphine is 1.88 mg/Kg ± 0.02. We calculated the opposite point of ED50 on the dose-response curve as downward point for morphine effect (4.3 mg/Kg). According to this calculation, 5 mg/Kg of morphine was chosen as downward point for the rest of the experiments (Figure 1). On the other hand, the number of compartment entering, locomotion, rearing and sniffing is reduced while the dose of morphine is increased (i.e. a clear drug-seeking behavior) (Table 1, [F (5, 38) = 1.343, p < 0.1] for sniffing, [F (5, 38) = 1.003, p < 0.1] rearing, [F (5, 38) = 0.992, p < 0.1] locomotion, and [F (5, 38) = 1.21, p < 0.1] compartment entering).

**Table 1:** Effects of different doses of morphine on the mesolimbic dopamine system related behaviors (number of sniffing, rearing and locomotion) and total compartment entering in rats on the test day. Data are shown as mean ± SEM for 6-8 rats

**Morphine (mg/Kg)**	**Sniffing ** **(No./10 min)**	**Rearing ** **(No./10 min)**	**Locomotion ** **(No./10 min)**	**Compartment entering ** **(No./10 min)**
0	11 ± 3	8 ± 2	12 ± 3	7 ± 3
1	14 ± 4	13 ± 3	18 ± 5	11 ± 4
2.5	12 ± 3	10 ± 4	14 ± 4	11 ± 3
5	7 ± 4	7 ± 3	12 ± 4	7 ± 3
7.5	9 ± 3	8 ± 3	9 ± 4	8 ± 3

*Effects of different doses of naloxone on the expression of downward morphine dose*

**Figure 2:**
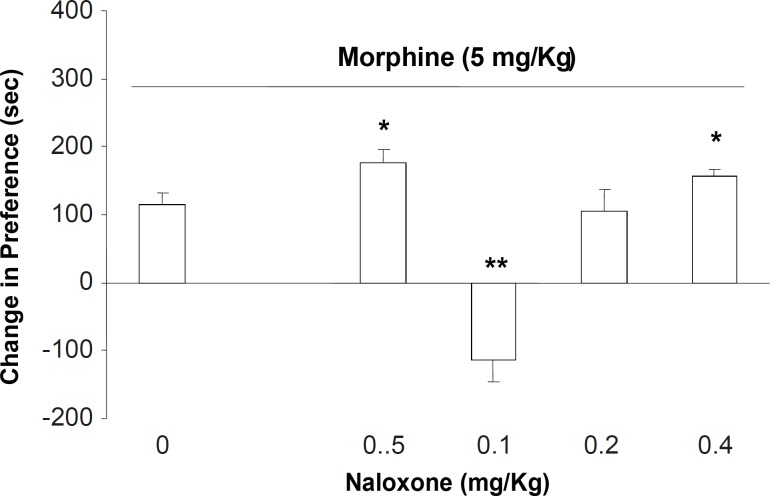
The effects of different doses of naloxone (0.05, 0.1, 0.2 and 0.4 mg/Kg; SC.) on the expression of morphine-induced place preference when challenged with the downward point dose of morphine (5 mg/Kg). Data are shown as mean ± SEM for 6-8 rats, *p < 0.05, **p < 0.01 different from the saline control group

In the second part of the experiments, different doses of naloxone (0.05, 0.1, 0.2 and 0.4 mg/Kg; s.c.) were administered to the animals which received downward morphine (5 mg/Kg; s.c.) in the conditioning days. Our results showed that the administration of naloxone for inhibition of opioid receptors on the expression day naloxone in doses of 0.05 and 0.4 mg/Kg increased the expression of morphine place preference (Figure 2, [F (5, 39) = 6.78, p < 0.0001]). However, the antagonist at dose of 0.1 mg/Kg induced a significant place aversion instead of place preference (Figure 2). Measurement of sniffing, rearing, locomotion and compartment entering also showed that these factors did not changed among the groups (Table 2, [F(5, 39) = 0.56, p < 0.1] for sniffing, [F(5, 39) = 0.988, p < 0.1] rearing, [F (5, 39) = 1.87, p < 0.1] locomotion, and [F(5, 39) = 0.99, p < 0.1] compartment entering).

**Table 2:** Effects of different doses of naloxone (0.05, 0.1, 0.2, 0.4 mg/Kg; SC) on the mesolimbic dopamine system-related behaviors (number of sniffing, rearing and locomotion) and total compartment entering in rats on the test day. Data are shown as mean ± SEM for 6-8 rats.

**Naloxone (mg/Kg)**	**Sniffing** **(No./10 min)**	**Rearing** **(No./10 min)**	**Locomotion** **(No./10 min)**	**Compartment entering** **(No./10 min)**
0	14 ± 3	11 ± 2	11 ± 3	14 ± 3
0.05	11 ± 3	10 ± 4	15 ± 6	12 ± 4
0.1	11 ± 3	11 ± 4	12 ± 4	13 ± 5
0.2	12 ± 3	12 ± 4	12 ± 3	14 ± 3
0.4	11 ± 3	13 ± 3	12 ± 3	14 ± 4

## Discussion

Our results indicated that the inhibition of opioid receptors by naloxone could potentiate the expression of morphine-induced place preference conditioning in doses of 0.05 and 0.4 and reverses the opioid effect in dose of 0.1 mg/Kg. It is important to notify that the similar results obtained for low and high doses of naloxone may not have similar base and mechanism(s). On the other hand, numbers of sniffing, rearing, and locomotion as indicators of mesolimbic dopamine-system activity (15-20) were not different form the control group. Results obtained from our experiments indicated that naloxone in low and high doses exacerbate and in medium dose (*e.g.*, 0.1 mg/Kg) reversed the expression of morphine CPP. These findings indicated the involvement of opioid receptors in the phenomenon. In agreement with previous studies, our results indicated that morphine can induce place preference in rats and mice (For rev see: 6, 21, 22). However, our results indicated that the maximum effect on place preference was achieved in dose of 2.5 mg/Kg which is also reported in previous studies (6). Previous studies also indicated that opioid receptor inhibition reduced the acquisition and expression of place preference induced by opioids (6, 23, 24, 25, 26, 27). On the other hand, in the previous studies, the effects of antagonists were observed on the doses of opioids which induced maximum response (6, 24, 27); this response was achieved at doses in which probably just one type of the opioid receptors was occupied by the opioid (6, 13, 24), though, in the present study, we proposed that by blockade of opioid receptors when downward shift of morphine dose-response curve was begun, dissociation the influence of different opioid receptors could be achieved on the morphine place preference. The special point here is that the receptor shifting may be occurring. In agreement with our proposal, studies have shown that agonists of kappa opioid receptors can induce place aversion in mice (6, 23, 27). It would have been quite normal that if these receptors were inhibited (by naloxone for example), the effects of other opioid receptors could have been observed. As the rewarding effects of opioids are related to mu-opioid receptor activation (23), in the present study, one can conclude that inhibition of the other opioid receptors is the main reason for augmentation of the expression of morphine-induced place preference which was observed for high naloxone (0.4 mg/Kg) dose. It seems that the reason of potentiating the expression of morphine-induced place preference by low dose of naloxone (*e.g. *0.05 mg/Kg) is different and needs another explanation. However, it is now clear that mu-opioid receptors can either inhibit or activate the enzyme adenylate cyclase (AC) in the plasma membrane of the target cells (13, 28), which in turn, decreases or increases intra-cellular concentration of the cyclic adenosine monophosphate (cAMP), respectively (13, 28). Although the population of the last kind of receptors is much lower, activation of this kind of opioid receptors can unfortunately mask the real potential of the seconds (28). Therefore, inhibition of these receptors by low doses of opioid receptor antagonists can increase morphine antinociception as well as reward potency (13, 29, 30). Our explanation for the effect of low dose of naloxone is that the drug may inhibit the mu-opioid receptors which activate AC and unmasks the real role of the main mu-opioid receptor population which inhibit AC (13). So, the potentiation of the expression of morphine-induced place preference was observed as a result.

One important finding from our results is that the effects were not due to a change in dopamine-mesolimbic system activity as the behaviors related to the activities of mesolimbic dopamine system (dopamine related behaviors) such as sniffing, rearing and locomotion did not changed for the experimental and control groups. In addition, total compartment entering as an indicator of decision making was not changed which indicated that the behaviors related (at least in part) to the other central nervous system regions (i.e. frontal cortex) was not affected by our intervention. 

In conclusion, our results indicated that the expression of morphine place preference may be related to the mu-opioid receptors activity. Besides, the receptors may also be similar to opioid receptors that inhibit AC rather than those activate the enzyme. 
